# Determining the role of basophil activation testing in reported type 1 allergy to beta-lactam antibiotics

**DOI:** 10.3389/falgy.2024.1512875

**Published:** 2024-12-24

**Authors:** Markus Reitmajer, Antonia Strauss, Christian Klinger, Maximiliane Maaß, Wolfgang E. Kempf, Joerg Fischer, Manfred Kneilling, Sebastian Volc

**Affiliations:** ^1^ Department of Dermatology, Eberhard Karls University, Tuebingen, Germany; ^2^ FACS Core Facility Location Tal, Department of Dermatology, Eberhard Karls University, Tuebingen, Germany; ^3^ Department of Dermatology and Allergology, University Augsburg, Augsburg, Germany; ^4^ Department of Preclinical Imaging and Radiopharmacy, Werner Siemens Imaging Center, Eberhard Karls University, Tuebingen, Germany

**Keywords:** beta-lactam-antibiotic, basophil activation test, type 1 allergy, hypersensitivity, delabeling

## Abstract

**Background:**

Allergy to beta-lactam antibiotics (BLA), especially to penicillin, is the most commonly reported drug allergy by patients. Alternative antibiotics can yield negative consequences, such as extended hospitalization days due to less efficacy and overall higher costs. The basophil activation test (BAT) is an *in vitro* assay, in which activation of an individual's own basophils is quantified by flow cytometry. It is an increasingly applied *in vitro* method in allergy testing that is also gaining traction in drug allergies.

**Methods:**

We correlated 37 BAT results with skin test results. The cohort exclusively included patients with suspected type I BLA allergy. In addition, we examined the concordance of these results with clinical symptoms reported in the BLA patients’ medical histories.

**Results:**

BLA-BAT revealed a high specificity of 92.3% [95% confidence interval (CI) 66.7–98.6] but a low sensitivity of only 20.8% (95% CI 9.24–40.47) using BLA-skin tests as a comparator. Negative BLA-BAT in patients with a history of grade I anaphylaxis yielded doubt on the assumption of grading. The exclusion of grade I BLA anaphylaxis increased the sensitivity to 29.4% (95% CI 13.28–53.13) with a still high specificity of 85.7% (95% CI 48.69–97.43). When ImmunoCAP was available, we compared specific IgE and BAT results by using Cohens' kappa (κ) and revealed a moderate level of agreement (κ = 0.538, *p* = 0.029).

**Conclusion:**

BAT reveals specific positive results exclusively in patients with cephalosporin anaphylaxis. However, these findings could not be generally confirmed in the heterogeneous group of BLA.

## Introduction

### Background

Allergy to beta-lactam antibiotics (BLA), especially penicillin allergy, is the most frequent self-reported drug allergy, with a prevalence of approximately 10%. For diagnostic as well as treatment reasons, it is essential to differentiate between allergic and non-allergic patients. However, large-scale studies show that 80%–95% of these patients are found not to be allergic after extensive diagnostic workup (1–3).

The diagnosis of an allergy to BLA and subsequent usage of alternative antibiotics correlates with extended hospitalization days due to less effective and potentially more harmful medications, higher-priced second-line therapies, and the increased risk of hospital acquired infections as well as further development of microbial resistances to antibiotics [*Clostridium difficile*, methicillin-resistant *Staphylococcus aureus* (MRSA), and vancomycin-resistant *Enterococcus* (VRE)]. All of this adds up to a higher burden for the healthcare system (4). Considering the broad usage and indispensable role of BLA in a multitude of therapeutical indications, it is important to verify the suspected medication as the causative allergen or to remove the given “allergic” label. Despite their risk of allergic reactions, BLA are associated with fewer side effects or drug interactions than other comparable antibiotics, e.g., fluoroquinolones (5).

Clinical evaluation, including a detailed description of the symptoms, can be obtained from the patients themselves (6, 7). Based on medical history, patients can be stratified into grades I–IV according to the Ring and Messmer anaphylaxis classification (8). Depending on the medical history, *in vivo* skin test methods are the next step. When skin and *in vitro* testing yield negative results, the gold standard to verify IgE-mediated BLA is a provocation test with the culprit drug. However, disadvantages of serious adverse reactions during *in vivo* testing procedures have to be considered (7, 9–11). A considerable alternative would be *in vitro* testing, which has recently undergone notable progress. The quantification of specific IgE (sIgE) antibodies could be indicated, for example with the ImmunoCap® system (Phadia®) and the basophil activation test (BAT). The BAT (*ex vivo* assay), in which activation of individual's own basophils is quantified *in vitro* by flow cytometry, has emerged as an alternative predictive tool, but is still in the process of becoming established and not yet frequently used in the routine assessment of BLA allergy (supplementary tool). It is a functional assay that correlates with histamine release and measures the degree of degranulation after stimulation with allergen by flow cytometry (7, 12–14).

### Objective

The aim of this retrospective analysis was to define the role of BAT in type 1 allergy to BLA. We tested 34 patients with a suspected type 1 allergy to one or several BLAs via BAT and calculated sensitivity, specificity, and positive/negative predictive values (PPV/NPV), using skin tests as the gold standard. In accordance with our established diagnostic protocol, we also correlated clinical data, encompassing symptoms and grading according to Ring and Messmer (8). Herein, we focus on uncovering the potential role and abilities of BAT in the diagnostic procedure of BLA allergy testing.

## Methods

### Subjects and diagnostic workflow

A total of 34 patients were included in the survey and underwent BAT for a suspected type 1 allergy to BLA between 2007 and 2022 at the Department of Dermatology, Eberhard Karls University, Tuebingen, Germany. Findings were obtained as part of an individual diagnostic process and not as part of a clinical study. In our established diagnostic procedure, it is common to use further advanced diagnostics, such as the BAT. The reason for this practice is the potential risk of a false-positive reaction due to the known irritant effects of the skin tests.

The inclusion criterion for all retrospectively analyzed patients was a detailed clinical history regarding potential type 1 allergy symptoms. The data are based on a careful anamnesis and pre-existing medical documentation of the symptoms. The severity of the anaphylactic symptoms was classified according to the Ring and Messmer classification (8). Patients without a high-risk group stratification of adverse reactions underwent additional skin testing.

This retrospective analysis was performed in accordance with the local ethical committee of the Medical Faculty of the Eberhard Karls University, Tuebingen, Germany, and the general recommendations outlined in the Declaration of Helsinki.

### Skin tests

Prick and intradermal tests (IDT) were performed according to the recommendations of the Global Allergy and Asthma European Network GA2LEN (15). Histamine was used as a positive control for prick testing in a concentration of 10 mg/ml and for IDT in a concentration of 0.1 mg/ml. NaCl 0.9% was applied as a negative control. Prick and IDTs were considered positive, strictly in comparison to the positive and negative control.

### *In vitro* tests

#### Specific immunoglobulin E

Antigen sIgE in the sera of patients was determined using the automated ELISA System ImmunoCap 250 (Thermo Fisher Phadia, Freiburg, Germany), according to the manufacturer's instructions. CAPs were commercially available for penicillin G, penicillin V, amoxicillin, ampicillin, and cefaclor [but not for cefuroxime, ceftriaxone, cefazolin, meropenem, and penicilloyl-poly-lysine (PPL)]. sIgE in the patient serum binds to the allergen of interest covalently linked to the ImmunoCAP. Detection is carried out through enzyme-linked antibodies against IgE and a fluorogenic developer reagent. Concentrations were calculated using a calibration curve. Sensitization to the antigen was considered positive at values >0.35 kU/L.

#### Basophil activation test

Allergen-induced basophil activation in ethylenediaminetetraacetic acid (EDTA) blood was determined using the commercially available BAT Flow2 CAST® (Bühlmann Laboratories AG, Schönenbuch, Switzerland) according to the manufacturer's instructions (16). Available commercial allergens used were penicillin G, penicillin V, ampicillin, amoxicillin, cefaclor, ceftriaxone, cefazolin, cefuroxime, meropenem, and PPL (Bühlmann Laboratories AG, Schönenbuch, Switzerland). Allergens were tested in the concentration recommended by the manufacturer plus four serial 1:5 dilutions (to determine the strongest possible activation level). EDTA anti-coagulated venous blood was incubated with allergen, stimulation controls [anti-Fc*ε*RI Ab and N-formylmethionine-leucyl-phenylalanine (fMLP)], with a provided stimulating solution as a negative control and antibody cocktail [anti-CD63 phycoerythrin (PE) and anti-IgE fluorescein isothiocyanate (FITC)] for 15 min at 37°C, followed by lysis of red blood cells. Surface presence of CD63 was determined on CCR3^+^, side scatter (SSC)^low^ basophils by flow cytometry (BD FACSCalibur; BD Biosciences, San Jose, CA, USA) using CellQuest™ software (BD Biosciences, San Jose, CA, USA). BAT was evaluable when at least one of the stimulation controls was >10% CD63-positive basophils. Basophil activation was indicated when the percentage of activated basophils was ≥5% (technical cutoff) and/or the stimulation index (SI) was ≥2. The SI was calculated as the quotient of the patient's background and allergen-activated basophils (Supplementary Figure S1).

### Statistical analysis

To evaluate BAT performance, we calculated the sensitivity, specificity, and PPV/NPV, in comparison with skin tests as gold standards. It is worth mentioning here that skin tests have limitations regarding their sensitivity and NPV (17–19). Concordance between sIgE and BAT results were calculated with Cohen's kappa (κ) statistics. For statistical analyses, we used SPSS Statistics version 28.0.0.0 (IBM Corp., Armonk, NY, USA). Diagrams were created with GraphPad PRISM 9.5.0 (Dotmatics, Boston, MA, USA).

## Results

We evaluated 34 patients [9 men (26.5%), 25 women (73.5%); mean age 50.68 ± 16.1 years). The patients’ characteristics and test results are shown in Table 1 for each patient in detail.

**Table 1:** Patients’ characteristics and detailed test results.

No. of patient	Sex	Age (years)	Symptoms	Anaphylaxis grade	Suspected substance (medical history)	Substance for testing	Prick	IDT	Result of skin test	sIgE	BAT
1	♂	52	C	I	BLA in general	Cefuroxime	−	−	−	n/a	−
2	♀	64	C	I	Amoxicillin	Amoxicillin	−	n/d	−	−	−
3	♂	52	C	I	Cefuroxime	Cefuroxime	+	−	+	n/a	−
4	♂	51	C	I	Cefuroxime	Cefuroxime	+	−	+	n/a	−
5	♀	27	C	I	Benzylpenicillin	Benzylpenicillin	−	+	+	−	−
6	♀	63	C	I	Meropenem	Meropenem	−	+	+	n/a	−
7	♀	23	C	I	Ampicillin	Ampicillin	−	+	+	−	−
8	♀	44	C	I	Amoxicillin	Amoxicillin	−	n/d	−	−	−
9	♀	69	C	I	Cefuroxime Phenoxymethylpenicillin	Cefuroxime	+	n/d	+	n/a	−
9		69				PPL	+	n/d	+	n/a	−
10	♀	34	C	I	Amoxicillin	Amoxicillin	−	n/d	−	−	−
11	♀	46	C	I	Amoxicillin	Amoxicillin	−	−	−	−	−
12	♀	16	C	I	Cefuroxime	Cefuroxime	−	−	−	n/a	−
13	♂	46	C, R	II	Phenoxymethylpenicillin	Phenoxymethypenicillin	−	n/d	−	+	−
14	♀	55	C, R	II	Benzylpenicillin, cephalosporins in general	Benzylpenicillin	−	n/d	−	−	−
14		55				Cefaclor	−	n/d	−	−	+
15	♂	49	C, G	II	Cefuroxime	Cefuroxime	+	+	+	n/a	−
16	♀	63	C, R	II	BLA in general	Cefaclor	−	n/d	−	−	−
16		63				Benzylpenicillin	−	+	+	+	−
17	♀	76	C, R	II	Cefuroxime	Cefuroxime	+	−	+	n/a	−
18	♂	43	C, R	II	Cefaclor	Cefaclor	+	n/d	+	+	+
19	♀	75	R	II	Cefazoline	Cefazoline	+	n/d	+	n/a	−
20	♀	45	C, R, CV, G	II	Cefuroxime	Cefuroxime	−	+	+	n/a	−
21	♂	52	C, R	II	Ceftriaxone	Ceftriaxone	−	−	−	n/a	−
22	♀	23	C, R	II	Phenoxymethylpenicillin	Phenoxymethylpenicillin	−	n/d	−	−	−
23	♀	74	C, R	II	Cefazoline	Cefazoline	−	+	+	n/a	−
24	♀	40	C, R	II	Cefuroxime	Cefuroxime	−	−	−	n/a	−
25	♀	61	C	II	Cefaclor	Cefaclor	+	n/d	+	+	+
26	♀	58	C, R	II	Cefuroxime	Cefuroxime	+	+	+	n/a	−
27	♀	70	C, R, CV	II	Cefuroxime	Cefuroxime	+	+	+	n/a	−
28	♀	40	C, CV	III	Cefuroxime	Cefuroxime	−	+	+	n/a	+
29	♀	56	G, R, CV	III	Cefaclor	Cefaclor	+	n/d	+	+	+
30	♂	61	C, CV	III	Cefuroxime	Cefuroxime	+	+	+	n/a	−
31	♀	19	C, CV	III	Cefuroxime	Cefuroxime	+	+	+	n/a	−
32	♂	54	C, R, CV	III	Cefuroxime	Cefuroxime	+	n/d	+	n/a	+
33	♀	59	C, R, CV, G	III	Cefuroxime	Cefuroxime	+	n/d	+	n/a	−
34	♀	63	C, R, CV	III	Amoxicillin	Amoxicillin	−	+	+	−	−

C, cutaneous; R, respiratory; CV, cardiovascular; G, gastrointestinal symptoms; n/a, not commercially available; n/d, not done; (−), negative test result; (+), positive test result; IDT, intradermal test; PPL, penicilloyl-poly-lysine.

The patient characteristics and detailed test results for each patient are shown. Anaphylaxis grade is according to the classification of Ring and Messmer.

### Patients demonstrated a high prevalence of cutaneous, respiratory, and cardiovascular symptoms

**Figure 1:**
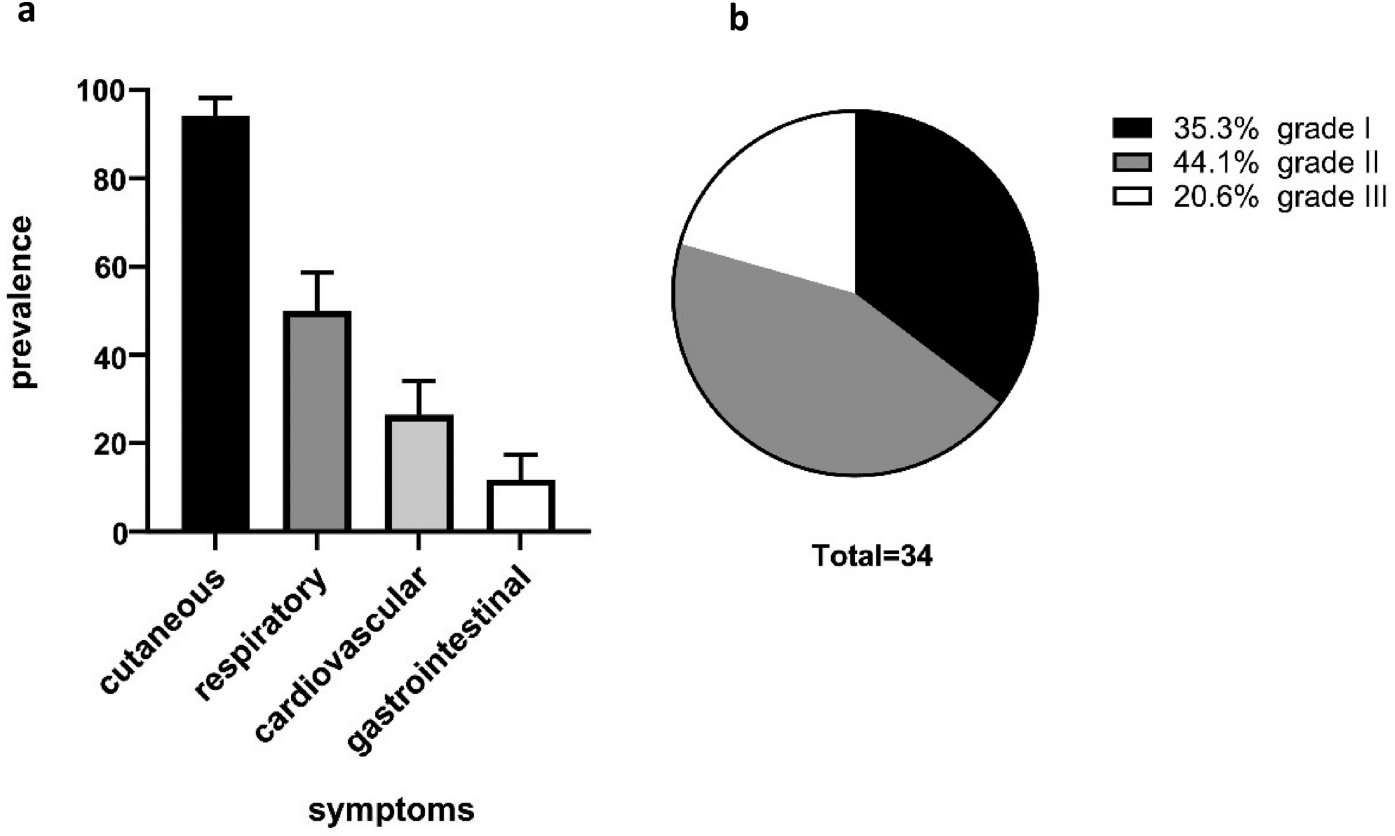
Patient characteristics. The cohort showed a high prevalence of cutaneous anaphylaxis symptoms (94.1%) followed by respiratory symptoms (50.0%) and cardiovascular symptoms (26.5%). Of our patients, 11.85% yielded gastrointestinal symptoms **(a)**. The severity grading of anaphylactic reactions was classified according to Ring and Messmer. A total of 12 (35.3%) patients exhibited grade I anaphylaxis reactions, 15 (44.1%) patients grade II anaphylaxis reaction, and 7 (20.6%) patients grade III anaphylaxis reaction **(b)**.

Patient documentation of anaphylactic symptoms demonstrated a high prevalence of cutaneous (94.1%), but also respiratory (50.0%) and cardiovascular reactions (26.5%). Only four (11.8%) participants exhibited gastrointestinal symptoms (Figure 1). In total, 12 (35.3%) patients developed a grade I reaction, 15 (44.1%) patients a grade II reaction, and 7 (20.6%) patients a grade III reaction (Figure 1), according to the Ring and Messmer classification.

### Skin tests proved to be safe and sensitive but not highly specific

In total, 37 prick and 21 IDTs were performed. No life-threatening anaphylactic complications were observed. Prick tests yielded positive results in 43.2% (*n* = 16/37), while IDT tests exhibited positive results in 61.9% (*n* = 13/21) of the conducted tests (Table 2). Skin testing revealed positive results for amoxicillin (*n* = 1/7), ampicillin (*n* = 1/2), cefaclor (*n* = 3/5), cefazolin (*n* = 2/3), cefuroxime (*n* = 18/29), meropenem (*n* = 1/2), penicillin G (*n* = 2/5), and PPL (*n* = 1/1) (Table 2). Skin tests exhibited positive results in 6 out of 12 patients, with a classified grade I anaphylactic reaction in the clinical history.

**Table 2:** Results of BAT, skin, and sIgE tests with beta-lactam antibiotics.

Results of skin tests with beta-lactam antibiotics					
Group	Substance	Skin tests		sIgE test *n* (%)	BAT *n* (%)
		Prick *n* (%)	IDT *n* (%)		
Penicillin	Amoxicillin	0/5	**1/2 (50%)**	0/5	0/5
	Ampicillin	0/1	**1/1 (100%)**	0/1	0/1
	Benzylpenicillin	0/3	**2/2 (100%)**	**1/3 (33%)**	0/3
	Phenoxymethylpenicillin	0/2	0/0	**1/2 (50%)**	0/2
Cephalosporin	Cefaclor	**3/5 (60%)**	0	**3/5 (60%)**	**4/5 (80%)**
	Cefazoline	**1/2 (50%)**	**1/1 (100%)**	0/0	0/2
	Ceftriaxone	0/1	0/1	0/0	0/1
	Cefuroxime	**11/16 (69%)**	**7/13 (54%)**	0/0	**2/16 (13%)**
Carbapenem	Meropenem	0/1	**1/1 (100%)**	0/0	0/1
	PPL	**1/1 (100%)**	0/0	0/0	0/1
	TOTAL	**16/37 (43%)**	**13/21 (62%)**	**5/16 (31%)**	**6/37 (16%)**

IDT, intradermal test; PPL, penicilloyl-poly-lysine; sIgE, specific IgE; BAT, basophil activation test.

Positive results are written in bold.

### sIgE correlates with grade II–III anaphylaxis

A total of 16 specific IgE tests were performed. We detected a specific IgE against cefaclor in three patients, which was confirmed by BAT. We identified a specific IgE against penicillin G in one patient and we found a specific IgE against penicillin V in one patient (Table 2). However, the results for penicillin V and penicillin G could not be verified via BAT. Positive sIgE results correlated with (respective) positive penicillin G and cefaclor skin test results but not with penicillin V skin testing. All positive sIgE results are related to patients with at least a grade II anaphylaxis reaction (Ring/Messmer classification).

### BAT shows positive results for the cephalosporins cefaclor and cefuroxime, confirming sIgE results with a moderate level of agreement

BAT was performed at least once in 34 patients. In some patients, BATs were performed with more than one BLA, resulting in a total of 37 tests (Table 2). BAT yielded positive results in four patients with cefaclor-related anaphylaxis and in two patients with cefuroxime-related anaphylaxis. When correlating positive BAT results with skin testing results, we determined positive results for cefaclor in three patients. In line with this, in these three patients, we were able to detect specific sIgE against cefaclor. The two patients with positive cefuroxime BAT also revealed positive skin testing results for cefuroxime. Unfortunately, no CE-certified sIgE testing was available for cefuroxime. The BAT revealed no positive results for the other two cephalosporins, cefazolin (0/2) and ceftriaxone (0/1).

Next, we observed the level of agreement of positive/negative results between the *in vitro* tests (ImmunoCap testing, BAT) using Cohen's kappa (κ). We revealed a moderate level of agreement between ImmunoCap and BAT results (κ = 0.538, *p* = 0.029, *n* = 16) according to the classification of Landis and Koch (20).

### No positive BAT results were found for grade I patients

BAT achieved a specificity of 92.3% [95% confidence interval (CI) 66.7–98.6, *n* = 37] and a sensitivity of 20.8% (95% CI 9.24–40.47, *n* = 37) when compared to skin testing. PPV was 83.3% (95% CI 44.6–99.0, *n* = 37) and NPV was 38.7% (95% CI 23.0–56.2, *n* = 37). Next, we correlated the grading according to Ring and Messmer for each patient. All positive BAT results were found in patients who experienced at least a grade II anaphylactic reaction. We excluded these patients to investigate the possibility of false-positive results due to inaccurate medical histories leading to a suspected grade I classification. This improved the sensitivity in this new group to 29.4% (95% CI 13.28–53.13, *n* = 24), with a still high specificity of 85.7% (95% CI 48.69–97.43, *n* = 24). PPV was 83.3% (95% CI 44.6–99.0, *n* = 24) and NPV was 33.3% (95% CI 14.8–56.3, *n* = 24). Although we performed the BAT with 12 different BLAs, we could exclusively obtain positive results with two substances (cefaclor and cefuroxime).

## Discussion

For allergy diagnosis, BAT demonstrates a broad spectrum of potential applications, including insect venoms, latex, food allergens, BLA, muscle relaxants, pyrazolones, and non-steroidal anti-inflammatory drugs (14, 21–23). In certain domains, the role of BAT is established, for instance, in the context of insect venom allergy (21, 24, 25). However, its role in medication allergies is currently undefined (25). Herein, we demonstrate the limitations of BAT in the realm of type 1 allergy to BLA.

The mean age and gender distribution in this retrospective analysis are consistent with those observed in other studies (12, 21, 26). BAT results revealed a high specificity of 92.3%, coinciding with existing studies (12, 27). However, the overall sensitivity of 20.8% was revealed to be slightly lower than in the reported literature, where values were in the range of 30%–55% (12, 13). Although the comparability of the existing studies and our data is not given due to different inclusion criteria, test protocols, and testing methods, we questioned the potential underlying reasons for the decreased sensitivity.

In the present study, BAT seems to be a recommendable tool in specific settings, such as higher grades of cephalosporin-induced anaphylaxis, but not advisable as an initial reliable diagnostic tool for broad BLA allergy type 1 diagnostics. Thus, our results yield a high sensitivity exclusively in BAT investigations of higher grade (≥II) anaphylaxis patients. We excluded these patients, hypothesizing that a false-positive medical history might result in a misclassification in grade I. The statistical analysis of the newly defined group yielded a sensitivity of 29.4% and a specificity of 85.7%. However, this illustrates the challenge and, at the same time, the importance of medical history, before conducting skin and blood tests. Apart from this, our results suggest a higher pre-test probability of positive BAT, in patients receiving cephalosporins. Although we performed BAT with 12 different substances, we could exclusively obtain positive results for cefaclor and cefuroxime (cephalosporins) but not for the subgroup of aminopenicillins and penicillin V/G. Consequently, our results indicate that BAT could be more appropriate for cephalosporins and might have a diagnostic gap for non-cephalosporin BLA. A similar observation was previously made by Torres et al. in 2004 (27). Herein, cephalosporin revealed a higher sensitivity compared to substances such as amoxicillin, ampicillin, and PPL (27). Due to the limited size of our dataset and the available data in the literature, further studies are necessary to verify this.

We questioned whether the direct measurement of sIgE, i.e., using the ImmunoCap system, could replace the use of BAT. We revealed a moderate level of agreement between the sIgE and BAT results (κ = 0. 538, *p* = 0.029) according to the classification of Landis and Koch (20). With a moderate level of agreement, sIgE and BAT are not substitutes for each other but rather should be used in combination to increase each other's sensitivity. This was particularly evident in the correlation between the sIgE and BAT results in the case of penicillin G and penicillin V, as BAT could not confirm a single positive result from the ImmunoCap test. Nonetheless, it remains uncertain whether the ImmunoCap test is yielding false-positive results or if the BAT test is generating false-negative results. It is also debatable whether the use of basophils is suitable for the detection of type 1 allergy to BLA; however, as tissue mast cells are problematic to collect, basophils are used as a replacement in testing. Our results point to the reliability of the BAT when used in patients with a history of at least grade 2 anaphylaxis caused by cephalosporins. Whether it is unreliable in a different context, such as base penicillin antibiotics and/or grade 1 anaphylaxis, remains to be seen and should be the context of further, preferably prospective, investigation.

The role of BAT in the identification of type 1 allergy to BLA remains unclear. Most importantly, our data reveal that neither the sIgE nor the BAT can replace *in vivo* skin testing and also cannot fulfill the function of a confirmation test. However, the advantage of *in vitro* testing in reducing the risk of adverse reactions should be taken into consideration (11). Here, we also provide first data indicating that pre-selection by clinical history and restriction to the substance to be tested could potentially increase the sensitivity. Thus, continuative studies are necessary to validate the utility of BAT in the diagnostic procedure of BLA allergy testing. With further standardization in an individualized manner, BAT may attain a specific role in the future testing of BLA type 1 allergy.

## Data Availability

The original contributions presented in the study are included in the article/Supplementary Material, further inquiries can be directed to the corresponding author.

## Supplementary material

Supplementary Figure S1 Quantification of CD63 upregulation in cefaclor-incubated basophils—a representative positive example of a female patient. The upregulation of membranous CD63 by activated basophils was quantified as percentage of CD63-positive cells compared to the total number of basophils gated in R1. BAT was considered positive when a ≥5% cutoff value for positive basophils and an SI ≥2 was reached. Anti-Fc*ε*RI Ab and fMLP were used as stimulation/positive controls and the probe prepared with only stimulation buffer was used as a background/negative control. UL, upper left; UR, upper right; LL, lower left; LR, lower right.
