# Supplementary material for: Determining the role of basophil activation testing in reported type 1 allergy to beta-lactam antibiotics
Source: Front Allergy. 2024 Dec 24;5:1512875. doi: 10.3389/falgy.2024.1512875 (PMC11703728; doi:10.3389/falgy.2024.1512875)
Supplement: Supplementary Figure S1 — Quantification of CD63 upregulation in cefaclor-incubated basophils—a representative positive example of a female patient. The upregulation of membranous CD63 by activated basophils was quantified as percentage of CD63-positive cells compared to the total number of basophils gated in R1. BAT was considered positive when a ≥5% cutoff value for positive basophils and an SI ≥2 was reached. Anti-FcεRI Ab and fMLP were used as stimulation/positive controls and the probe prepared with only stimulation buffer was used as a background/negative control. UL, upper left; UR, upper right; LL, lower left; LR, lower right. [file Datasheet1.docx]

# Supplementary Material

## Supplementary Figure

|  |  | **background** | **anti-FceRI Ab** | **fMLP** |
| --- | --- | --- | --- | --- |


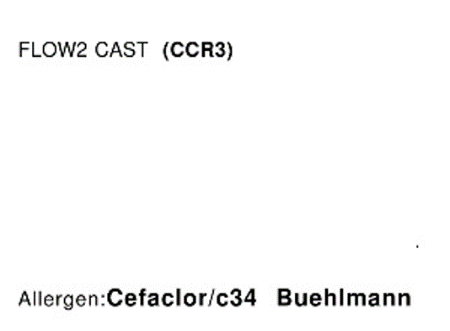

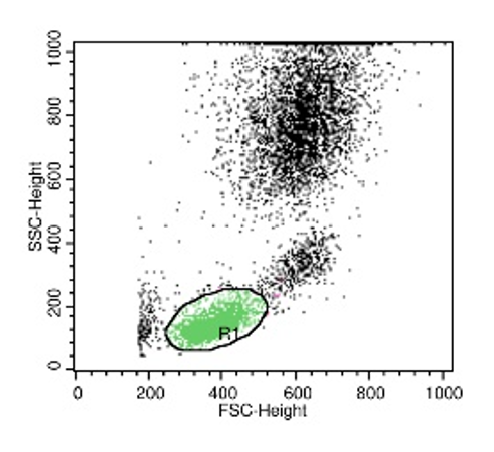

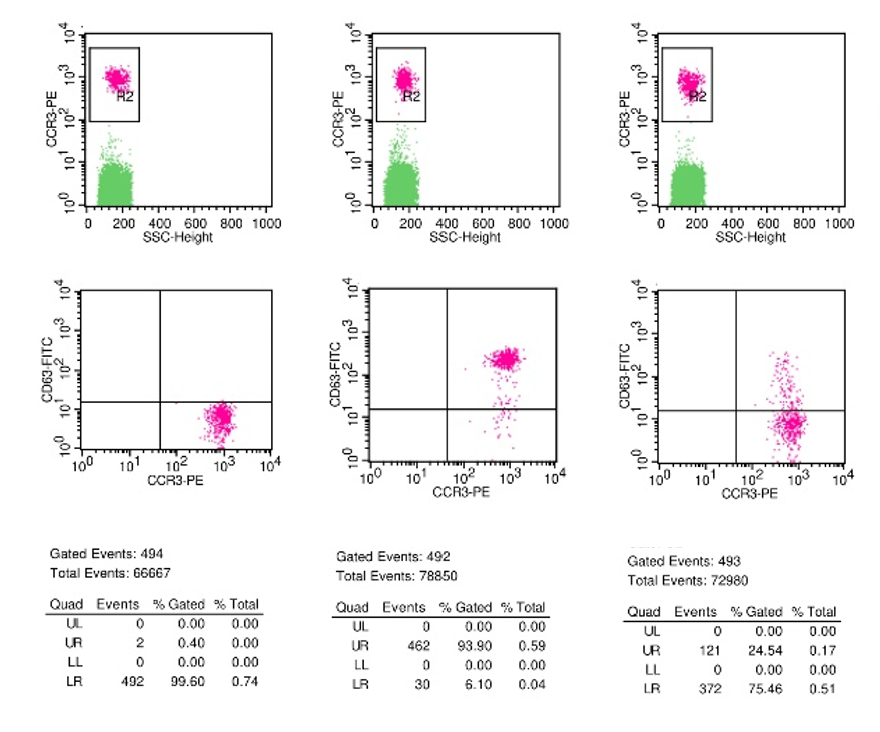


| **682 ug/ml** | **136,4 ug/ml** | **27,3 ug/ml** | **5,46 ug/ml** | **1,08 ug/ml** |
| --- | --- | --- | --- | --- |


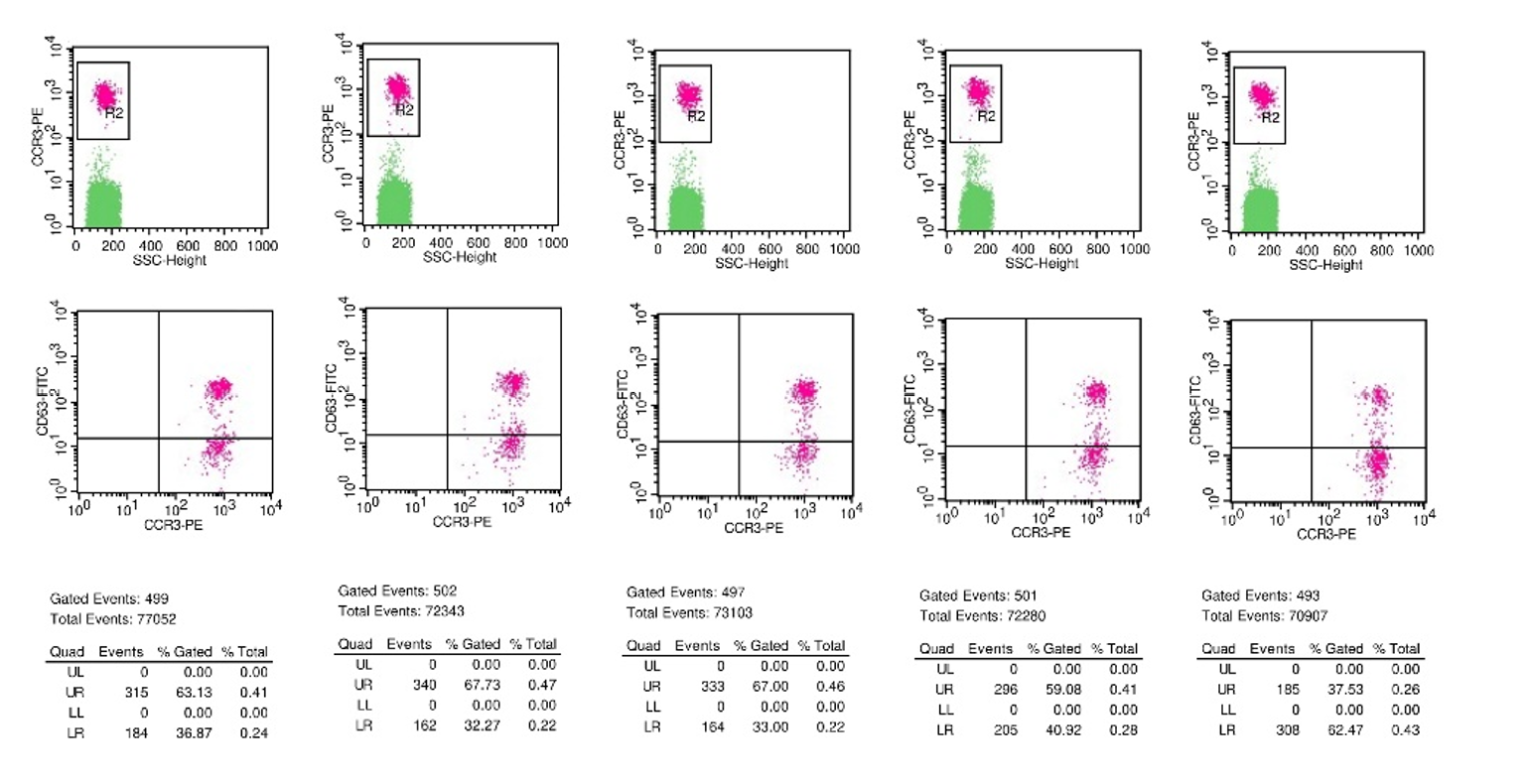


Supplementary Figure 1 Quantification of CD63 up-regulation in cefaclor-incubated basophils – a representative positive example of a female patient. The up-regulation of membranous CD63 by activated basophils was quantified as percentage of CD63 positive cells compared to the total number of basophils gated in R1. BAT was considered positive when a ≥ 5 % cut-off value for positive basophils and a stimulation-index (SI) ≥ 2 was reached. Anti-FcεRI Ab and fMLP were used as stimulation/positive controls and the probe only prepared with stimulation-buffer was used as background/negative control. The following abbreviations are used: upper left (UL), upper right (UR), lower left (LL), lower right (LR).
